# Solar radiation shapes viral ecology in an alpine lake at 4700 meters

**DOI:** 10.1093/ismeco/ycaf129

**Published:** 2025-08-08

**Authors:** Lin Zang, Yongqin Liu, Nianzhi Jiao, Lanlan Cai, Wei Wei, Xiaowei Chen, Yuying Chen, Keshao Liu, Rui Zhang

**Affiliations:** State Key Laboratory of Tibetan Plateau Earth System, Environment and Resources (TPESER), Institute of Tibetan Plateau Research, Chinese Academy of Sciences, Beijing, Beijing 100101, China; University of Chinese Academy of Sciences, Beijing, Beijing 100101, China; State Key Laboratory of Tibetan Plateau Earth System, Environment and Resources (TPESER), Institute of Tibetan Plateau Research, Chinese Academy of Sciences, Beijing, Beijing 100101, China; University of Chinese Academy of Sciences, Beijing, Beijing 100101, China; Center for the Pan-Third Pole Environment, Lanzhou University, Lanzhou, Gansu 730000, China; State Key Laboratory of Marine Environmental Science, Fujian Key Laboratory of Marine Carbon Sequestration, College of Ocean and Earth Sciences, Xiamen University, Xiamen, Fujian 361005, China; Earth, Ocean and Atmospheric Sciences Thrust, The Hong Kong University of Science and Technology (Guangzhou), Guangzhou, Guangdong 511453, China; Research Center for Environmental Ecology and Engineering, School of Environmental Ecology and Biological Engineering, Wuhan Institute of Technology, Wuhan, Hubei 430205, China; State Key Laboratory of Marine Environmental Science, Fujian Key Laboratory of Marine Carbon Sequestration, College of Ocean and Earth Sciences, Xiamen University, Xiamen, Fujian 361005, China; Center for the Pan-Third Pole Environment, Lanzhou University, Lanzhou, Gansu 730000, China; State Key Laboratory of Tibetan Plateau Earth System, Environment and Resources (TPESER), Institute of Tibetan Plateau Research, Chinese Academy of Sciences, Beijing, Beijing 100101, China; Archaeal Biology Center, Synthetic Biology Research Center, Shenzhen Key Laboratory of Marine Microbiome Engineering, Key Laboratory of Marine Microbiome Engineering of Guangdong Higher Education Institutes, Institute for Advanced Study, Shenzhen University, Shenzhen, Guangdong 518055, China

**Keywords:** viral ecology, high mountain lake, community composition, solar radiation, diel variation, vertical variation

## Abstract

Solar radiation plays a pivotal role in shaping viral ecology, fundamentally influencing ecological processes and biogeochemical cycles in aquatic environments. However, the dynamics of viruses in high-altitude lakes with intense solar radiation remain largely unexplored, hindering our understanding of their ecological significance. This study investigates diel and vertical variations in viral abundance, activity, diversity, and community in Lake Nam Co at an altitude of 4700 meters. We assess the effects of solar radiation on viral dynamics in the field through parallel light-transmitting and dark incubation experiments. Our findings reveal that intense solar radiation alters viral life cycles by extending latent periods, promoting lysogenic cycles, and accelerating degradation. Diel variations in viral dynamics are shaped by physicochemical shifts, particularly turbidity and pH changes driven by stream inflows, which buffer the effects of solar radiation and obscure clear diel patterns. Conversely, vertical variations in viral dynamics and community composition are predominantly dictated by solar exposure. This research represents the first comprehensive exploration of viral ecology in a high-altitude lake, significantly advancing our understanding of virus-mediated processes in biogeochemical cycling across alpine lake systems globally.

## Introduction

Viruses are the most abundant biological entities on Earth, fundamentally shaping ecological and biogeochemical processes [1–3]. Infecting nearly all aquatic organisms, viruses influence nutrient and energy cycles in the aquatic environment, acting as a major driver of biogeochemical fluxes [4]. Through lytic infection, viruses restructure microbial communities by converting biomass into dissolved and particulate organic matter, which disrupts energy and nutrient flow to higher trophic levels [1, 5]. In addition to lysis, lysogenic infections facilitate horizontal gene transfer and alter host physiology, indirectly affecting biogeochemical processes [6]. Viral degradation also supplies bioavailable organic matter into dissolved organic matter pools, enhancing their geochemical potential [7, 8]. Consequently, viruses are pivotal ecological players in the structuring and functioning of aquatic ecosystems.

Unravelling viral roles within aquatic ecosystems, including the environmental controls on viral dynamics and virus-host interactions, is increasingly critical. Viral ecology is governed by a complex interplay of abiotic and biotic factors [9]. Studies have revealed significant temporal, vertical, and spatial variation in viral dynamics and virus-host interactions in lakes, with influences from factors like chlorophyll-*a* (Chl*-a*), dissolved organic carbon (DOC), and bacterial activity [10, 11]. However, gaps remain in our understanding of virus dynamics, particularly in alpine lakes. Alpine lakes, important sentinels of climate change [12], experience unique environmental pressures, including extended ice cover, low water temperature, high UV radiation, and limited nutrient availability [13]. These conditions lead to simplified food webs [14], potentially amplifying the ecological role of viruses in alpine lake systems [15]. To address these knowledge gaps, detailed investigation into viral ecology in alpine lakes is required.

The Tibetan Plateau, known as “the third pole”, spans 5 million km^2^ with an average elevation of 4000 m above sea level [16]. The region’s clear and thin atmosphere results in minimal scattering of UV radiation, making it the highest solar radiation area in China [17]. Home to the world's largest concentration of alpine lakes, with 32 843 lakes covering 43 151 km^2^ [18], the Tibetan Plateau offers a unique environment to study freshwater viral ecology under extreme solar radiation. Given the importance of solar radiation for viral inactivation and decay, we hypothesize that solar radiation is a key driver of viral dynamics in these alpine lakes. To test this, we conducted a comprehensive study in Lake Nam Co, the second-largest alpine lake on the Plateau. Our work reveals diel and vertical variations in viral abundance, activity, life strategies, diversity, decay, and virus-host interactions, identifying key factors influencing viral dynamics in alpine lake ecosystems on the Tibetan Plateau.

## Materials and methods

### Site description

Lake Nam Co, the second largest lake in Tibet, situated in the central region of the Tibetan Plateau (30°30′–30°55'N, 90°16′–91°03′E, 4718 m a.s.l.) (Fig. S1) within the monsoonal transition zone between semi-humid and semi-arid areas [19]. The lake occupies a closed basin without surface outflow, mainly sustained by precipitation and meltwater inflow. Solar radiation in the entire catchment of Lake Nam Co is strong, and sunlight hours are long, with an average of 2880.9 sunlight hours annually [20]. Bathymetric survey revealed that nearly 50% of the lake area boasts a water depth exceeding 90 m [20]. Lake Nam Co is dimictic lake with complete mixing in the central part during winter and stratification form June to November [21].

### Field investigation and sample collection

Water samples were collected from both the shore and the euphotic (0.5 m) and aphotic (60 m) zones at the center of Lake Nam Co [22] (Fig. S1). Field investigations were conducted at the shore to assess diel variations in viral ecology, with sampling intervals every 3 hours from 7:00 on July 10^th^ to 7:00 on July 13^th^ (Fig. S2A). The exact times of sunrise (7:05) and sunset (21:03) were calculated using the astronomical software “*Astral*” (v3.0). Additional samples were taken from the euphotic and aphotic layers to evaluate vertical variation.

In situ geochemical parameters, including temperature, salinity, pH, Chl*-a*, dissolved oxygen (DO), and turbidity, were recorded using a YSI multiprobe Water Quality Sonde (YSI EXO2, Yellow Springs, OH, USA). Simultaneously, photosynthetically active radiation (PAR) was measured with a GER1500 radiometer (Spectra Vista Corporation, USA).

Approximately 100 ml of lake water was filtered through a 0.45 μm sterile syringe filter (Millipore, PVDF, 33 mm, SLHVR33RB, United States) to determine concentrations of major ions, DOC, and total nitrogen (TN). The filtrates were stored in 120-ml brown glass bottles at −20°C until analysis. In the laboratory, DOC and TN concentrations were quantified using a total organic carbon analyzer (TOC-Vcph, Shimadzu Corp, Japan). Major cations (Ca^2+^, Mg^2+^, K^+^, Na^+^) were analyzed using the Dionex™ ICS-2500 Ion Chromatography system, while major anions (Cl^−^, NO_3_^−^, SO_4_^2−^) were determined via Dionex™ ICS-2000 Ion Chromatography system (Dionex Company).

For biological analysis, samples were prefiltered through 20-μm mesh filters to remove larger particles and zooplankton. Subsequently, aliquots of 2 ml for bacterial and virus-like particle (VLP) abundance were fixed with glutaraldehyde (0.5% final concentration) for 15 minutes in the dark, flash-frozen in liquid nitrogen, and stored at −80°C to maintain sample integrity for subsequent analysis. Additionally, four 1.5 ml aliquots were immediately transferred to sterile 2 ml centrifuge tubes for in-field bacterial production measurements, as further detailed below.

An additional 200 L of water was sampled from euphotic and aphotic layer for metagenomic analysis. After removing phytoplankton and zooplankton using a 3 μm pore-size filter, the water was then concentrated using a large-scale tangential flow filtration (TFF) system with a 0.22 μm pore-size cartridge until the bacterial concentratevolume reached 2 L. The bacterial concentrates were subsequently stored in the dark at 4°C.

### Bacterial and virus-like particle counting

Bacterial and VLP abundance were quantified via flow cytometry (Epics Altra II, Beckman Coulter, USA). Total bacteria abundance (BA) was determined by SYBR Green I staining (20 000 × original dilution, Molecular Probe, USA), while autotrophic bacteria (e.g. cyanobacteria) were enumerated without staining, following the protocol of Marie et al. [23]. Heterotrophic bacteria abundance (HBA) was calculated by subtracting autotrophic bacterial abundance (ABA) from BA. VLP abundance (VA) was assessed using the same flow cytometry after SYBR Green I staining and incubated at 80°C for 10 min in a thermostat water bath, as descripted previously [24]. Blank controls were included to exclude background noise and non-viral particles. Data were analyzed using FlowJo v10.5.3. VLPs were distinguished from bacteria based on forward scatter and SYBR Green I fluorescence (FITC channel), with VLPs displaying smaller sizes and lower fluorescence intensities. The virus-to-bacterium ratio (VBR) was calculated by dividing VA by BA.

### Determination of bacterial production

Bacterial production was measured by the incorporation of ^3^H-leucine, following a modified version of the method described by Kirchman [25]. Triplicate 1.5 ml lake water samples and one trichloroacetic acid (TCA)-killed control (5% final concentration) were added with 20 nM ^3^H-leucine (specific activity, 40–60 Ci mmol^−1^) and incubated for 3 hours in the dark at *in situ* temperature. Incorporation was halted by adding 5% TCA (final concentration). In the laboratory, samples were centrifuged at 12 000 g for 10 minutes. The supernatant was removed, and the remaining pellet was washed twice with 5% ice-cold TCA and rinsed with 80% ethanol. After air-drying, 0.5 ml of scintillation liquid was added to each tube, and the contents were briefly vortexed. The samples were left to sit for ~24 hours, after which radioactivity was measured using a liquid scintillation counter (280TR, Waltham, USA). The rate of ^3^H-leucine incorporation was determined as the average of triplicate measurements, corrected for the TCA-killed control.

### Assessment of viral production and viral decay rate

Viral production (VP) and viral decay rates (VD) were assessed at a consistent location on the shore of Lake Nam Co from July 10^th^ to July 13^th^, at eight specific time points: 9:00 and 21:00 on the first day; 9:00, 21:00, and 24:00 on the second day; and 9:00, 15:00, and 21:00 on the third day (Fig. S2A). Additionally, water samples were collected from both the euphotic layer (0.5 m) and the aphotic layer (60 m) to evaluate viral production and decay rate.

VP in lake water was determined using the reduction approach [26]. Briefly, ~600 ml of water sample underwent concentration through TFF using a 0.22 μm pore-size polyvinylidene difluoride cartridge (Millipore; Pellicon filter-cassette PXGVPPC50, United States), resulting in a 50 ml bacterial concentrate. The <0.22 μm filtrate was then further subjected to a 30 kDa polysulfone cartridge (Millipore; Pellicon filter-cassette PXB030A50, United States) to obtain viral concentrate and viral-free water. Subsequently, 50 ml of bacterial concentrate was mixed with 250 ml of virus-free water and distributed into 50 ml sterile centrifuge tubes wrapped in tin foil for incubation at *in situ* temperature in the dark for 12 hours. Three tubes were designated for measuring lytic viral production, while the remaining three, supplemented with mitomycin C (1 μg/ml), were used to assess lysogenic viral production. Subsamples (1 ml) for VLP and bacterial abundance were collected at 0 hour and every 3 hours, fixed with glutaraldehyde (0.5% final conc.), stored in liquid nitrogen, and counted using flow cytometry.

Calculation of lytic and lysogenic viral production was executed using the online program VIPCAL [27]. Lytic viral production was determined based on the rate of viral accumulation during the 12-hour incubation [26]. Lysogenic viral production was calculated as the difference in the viral increase between the mitomycin C-treated and untreated samples. Additionally, the frequency of lytic infection cells (FIC) and frequency of lysogenic infection cells (FLC) were calculated according to the following formulas:


$$ \mathrm{FIC}\ \left(\%\right)=\left[\left({\mathrm{VA}}_{\mathrm{lytic}\_\max }-{\mathrm{VA}}_{\mathrm{lytic}\_\min}\right)/\mathrm{BS}\right]/{\mathrm{BA}}_0\times 100 $$

$$ \mathrm{FLC}\ \left(\%\right)=\left[\left({\mathrm{VA}}_{\mathrm{lyso}\_\max }-{\mathrm{VA}}_{\mathrm{lyso}\_\min}\right)/\mathrm{BS}\right]/{\mathrm{BA}}_0\times 100 $$

where VA_lytic_max_ and VA_lytic_min_ are the highest and lowest viral abundances encountered during the lytic viral production incubation [28]. The VA_lyso_max_ and VA_lyso_min_ values were the greatest and smallest differences between the viral increase in the mitomycin C-treated and control tubes [29]. The burst size (BS) was calculated as BS = VP/∆BA, where ∆BA was estimated by the decrease in bacterial abundance during incubation [30]. The BA_0_ represents the bacterial abundance at 0 hour in the viral production incubation.

The viral decay rate was determined following the protocol of Noble and Fuhrman [3]. Briefly, ~500 ml of lake water was filtered through a 0.22 μm pore-size polyvinylidene difluoride cartridge (Millipore; Pellicon filter-cassette PXGVPPC50, United States) to eliminate prokaryotes and other particles larger than 0.22 μm. The resulting 150 ml filtrate was divided into three 50 ml sterile centrifuge tubes wrapped in tin foil and incubated at *in situ* temperature in the dark for 12 hours. Subsamples (1 ml) were taken at 3-hour intervals, and the abundance of VLPs was quantified following the aforementioned procedure.

The viral decay rate was calculated from the slope of a linear regression fitted to the natural logarithm-transformed VLP abundance decline over time. This viral decay rate, expressed as a percentage per hour, was calculated by multiplying the slope by 100 [3].

### Assessing the impact of solar radiation on viral dynamics

To elucidate the influence of solar radiation on viral dynamics, we conducted four experiments both on the shore of Lake Nam Co at eight specific time points and within the lake's euphotic and aphotic layers (see Fig. S2B): (i) bacterial concentrate + virus-free water with/without mitomycin C in Whirl-Pak bags (lytic/lysogenic VP under light-transmitting conditions); (ii) bacterial concentrate + virus-free water with/without mitomycin C in 50 ml sterile centrifuge tubes wrapped in tin foil (lytic/lysogenic VP under dark conditions); (iii) bacteria-free filtrate in Whirl-Pak bags (VD under light-transmitting conditions); (iv) bacteria-free filtrate in 50 ml sterile centrifuge tubes wrapped in tin foil (VD under dark conditions). All centrifuge tubes and Whirl-Pak bags were incubated at *in situ* temperature for 12 hours to measure lytic and lysogenic VP, as well as VD, according to the protocols described earlier.

### DNA extraction and microbial metagenomic sequencing

Metagenomic DNA extraction was performed on the bacterial concentrate. After treatment with proteinase K, EDTA (0.5 M) and 10% w/v SDS at 55°C for 3 hours, the total DNA and viral DNA were extracted by the phenol/chloroform/isoamyl alcohol method. DNA concentration was determined using a NanoDrop 2000 Spectrophotometer (Thermo Scientific, Waltham, MA, USA). Subsequently, sequencing libraries were constructed and sequenced on an Illumina NovaSeq 6000 platform using 2 × 150 bp paired-end chemistry at MAGIGENE Biotech Co., Ltd (Guangzhou, China).

### Metagenome data analysis

Raw reads were quality controlled using Trimmomatic v0.33 with default parameters to remove sequencing adapters and low-quality sequences. Clean reads for each sample were assembled individually using MEGAHIT v1.1.1 (parameters: –min-contig-len 500 –k-min 21 –k-max 141) [31]. Open reading frames (ORFs) in contigs longer than 500 bp were predicted using Prodigal (v2.6.3) [32]. All predicted ORFs were clustered by MMseq2 (v13.45111) with default parameters (−e 0.001 –min-seq-id 0.9 -c 0.80). The longest sequence in each cluster was selected as the representative sequence, thereby constructing a non-redundant gene catalog for all samples [33]. Clean reads were mapped to nonredundant ORFs by Salmon to obtain transcripts per million (TPM) abundance [34]. Additionally, the ORFs were compared against KEGG (Kyoto Encyclopedia of Genes and Genomes) database to obtain functional annotation information.

Microbial taxonomic composition was assessed using Kraken2 (v 2.1.2). First, quality-filtered reads were taxonomically classified with Kraken2 against a pre-built reference database [35]. Species-level abundance estimates were subsequently refined using Bracken [36], generating both detailed and summary abundance profiles for downstream analyses.

Metagenomic binning of contigs was performed using Metabat2 v2.2.15 and Maxbin2 v2.2.7, followed by refinement with DASTool (−search_engine diamond –score_threshold 0). To establish representative species-level, the genomes were clustered into 160 metagenome assembly genomes (MAGs) using dRep (v3.2.0) based on >30% aligned fraction and a genome-wide ANI threshold of 95% [37]. We used GTDB-Tk (v1.6.0) to taxonomically classify representative MAGs of all clusters with the GTDB release 202 [38, 39]. The abundance of MAGs was calculated by CoverM (v0.6.0) using the TPM method.

Contigs with lengths ≥10 kb were retained for viral identification, and viral contigs were identified using a combined approach incorporating VirSorter2 (v2.2.1; all group parameters) [40], VirFinder v1.1 [41], DeepVirFinder v1.0 [42], and VIBRANT v1.2.1 [43], all executed with default settings. Contigs were retained if they met at least one of the following thresholds: VirSorter2 score ≥ 0.5, DeepVirFinder or VirFinder score ≥ 0.9 with *P* < .05, or identified as viral contigs by VIBRANT. All putative viral contigs identified by these methods were merged. We then applied CheckV (v0.7.0) [44] to assess and control the quality of the viral sequences. Following the standard operating procedure recommended by VirSorter2 [40], any contigs containing host genes and without any viral gene detected were excluded. Species-level viral populations (vOTUs) were generated by clustering viral contigs at 95% ANI across 85% of the shorter contigs using CD-HIT (v4.8.1) [45].

GeNomad v1.7.6 (end-to-end) was used for taxonomic classification of each vOTUs using default parameters [46]. To complement this, BLASTn and BLASTp alignments were conducted against the IMG/VR v4 database [47]. For the BLASTn-based approach, vOTUs were assigned an IMG/VR v4 Viral Cluster ID or taxonomy if the top hits exhibited ≥90% identity and ≥ 75% alignment coverage. For BLASTp, vOTUs were assigned to a viral family if more than 50% of its proteins had a bit-score ≥ 50 and matched the same family [48]. The abundance of these vOTUs was determined using the “contigs” module of CoverM with default parameters.

For “*in situ*” host prediction, MAGs from Lake Nam Co were used as target host. Multiple in silico pipelines were employed, including CRISPR spacer matching [49], BLASTn-based genomic alignment [49], k-mer–based genomic matching (VirHostMatcher) [50], and tRNA match-based approach (tRNAscan-SE) [51].

Lysogenic viruses were identified through a combined approach using CheckV v0.7.0 and the identification of lysogenic marker genes. Firstly, vOTUs marked as proviruses were retained. Subsequently, the DRAMv pipeline was utilized to annotate the identified viral proteins. vOTUs containing lysogenic marker proteins, such as integrase, invertase, serine recombinase, and CI/Cro repressors, were also considered indicative of lysogenic viruses [48].

The DRAMv pipeline was employed to annotate auxiliary metabolic genes (AMGs) in vOTUs. Initially, all vOTUs were reprocessed with VirSorter2 with “prepfor-dramv” functions to screen contigs for performing DRAM-v annotation. Genes assigned “-M” or “-M” flag with “-E” and/or “K” and with auxiliary scores of 1, 2, and 3 identified as putative AMGs [52].

### Statistics analysis and figure generation

Statistical analyses were performed using R V3.4.1 [53]. Differences among groups were assessed with the wilcox.test (*wilcox.test* function in the R package “*stats*”). Dunn test (*dunnTest* function in the R package *FSA*) was applied for post hoc comparisons to pinpoint specific divergent samples. Statistical significance was determined with the *cldList* function (R package “*rcompanion*”) at a threshold of *P* < .05. Data visualization included Sankey plots generated with “*networkD*” package, Venn diagram using the “*VennDiagram*” package, and cluster heatmaps created with the “*pheatmap*” package. Figure formats were adjusted using Adobe Illustrator for clarity and presentation. Bar charts were created using GraphPad Prism 8.0.2 (GraphPad Software Inc., San Diego, CA, USA).

## Results

### Physicochemical characteristics and microbial abundance in Lake Nam Co

Physicochemical parameters, including Chl*-a*, DO, DOC, TN, and ions, displayed no significant diel patterns over the 3-day period along the lake shore (Fig. S3A, B, Table S1). In contrast, temperature and PAR exhibited pronounced diel fluctuations (Fig. S3C, Table S1), with both increasing sharply after sunrise at 7:00, peaking near 16:00, and gradually declining thereafter. PAR diminished to zero by 21:00, consistent with sunrise and sunset times calculated from Astral data. Both pH, salinity, and total dissolved solids (TDS) showed slight but consistent diel variations (Fig. S3D, E, Table S1): pH rose during the night, declining after 16:00, while salinity and TDS showed the reverse pattern, decreasing at night and rising before 16:00.

**Figure 1:**
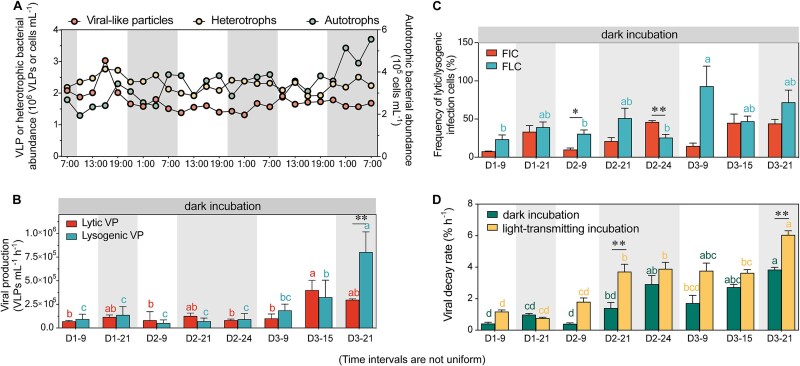
Diel variability in viral-like particle abundance, production, frequency of infected cells, as well as decay rate. (A) Diel variability in viral-like particle abundance, heterotrophic bacterial abundance, and autotrophic bacterial abundance. (B) Lytic and lysogenic viral production (VP) under dark incubations. (C) FIC and FLC under dark incubations. (D) Viral decay rates observed in dark and light-transmitting incubations.

Autotrophic bacterial abundance ranged from 1.9 × 10^5^ to 5.6 × 10^5^ cells ml^−1^, typically peaking around 7:00 before sunrise and generally rising at night (Fig. 1A). However, diel periodicity was not observed in viral-like particle abundance, heterotrophic bacterial abundance, virus-to-bacterium ratio, and heterotrophic bacterial production (Figs. 1A and S3F). VLP abundance ranged between 1.3 × 10^6^ and 2.1 × 10^6^ VLPs ml^−1^, while heterotrophic bacterial abundance remained stable between 1.9 × 10^6^ to 2.8 × 10^6^ cells ml^−1^ (Fig. 1A). Virus-to-bacterium ratio varied between 0.48 and 1.01. Spearman rank correlation analysis revealed that PAR had no significant effect on the abundance of viruses, heterotrophic bacteria, or autotrophic bacteria, but it significantly influenced the virus-to-bacterium ratio (Fig. S4). Notably, VLP abundance correlated significantly with Mg^2+^, K^+^, and SO_4_^2−^ concentrations, while HBA was influenced by lake turbidity. Autotrophic bacterial abundance showed sensitivity to changes in salinity and pH (Fig. S4).

### Diel pattern of viral ecology

At the lake shore, both lytic and lysogenic viral production exhibited no distinct day-night differences (dark incubations, Fig. 1B). Lytic production ranged from 6.9 ± 1.1 × 10^4^ to 4.0 ± 1.0 × 10^5^ VLPs ml^−1^ h^−1^, while lysogenic production varied between 5.2 ± 3.5 × 10^4^ and 8.0 ± 2.1 × 10^5^ VLPs ml^−1^ h^−1^. Except for a significantly higher lysogenic production at 21:00 on the third day, no notable differences between lytic and lysogenic production were detected (Fig. 1B). The FIC and FLC also lacked diel patterns, with FIC ranged from 7.6% to 46.0% and FLC from 23.3% to 92.7% (dark incubations, Fig. 1C). Viral decay rates varied between 0.4% and 3.8% and showed no diel periodicity (dark incubations, Fig. 1D).

### Vertical pattern of viral ecology

**Figure 2:**
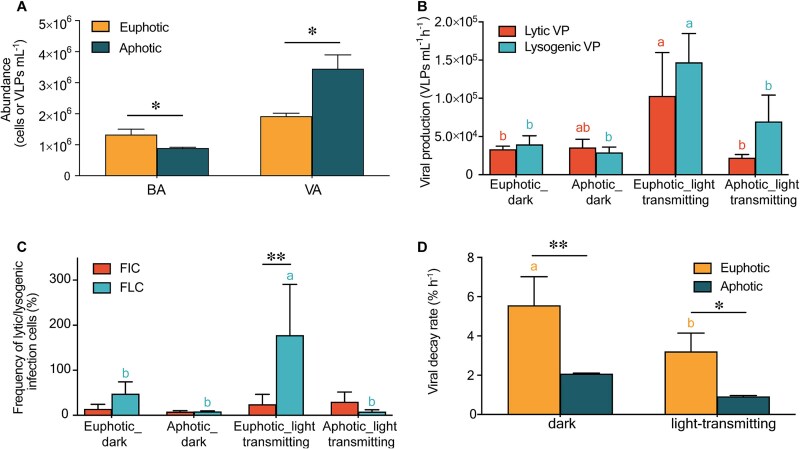
Viral abundance and dynamic parameters in euphotic and aphotic water layers. (A) Bacterial abundance (BA) and viral-like particle abundance (VA) in euphotic and aphotic layers. (B, C) Comparison of (B) lytic and lysogenic viral production and (C) FIC and FLC infected cells in euphotic and aphotic layers under dark and light-transmitting conditions. (D) Viral decay rates in euphotic and aphotic water layers under dark and light-transmitting incubations.

We also investigated vertical patterns in viral abundance, production, and decay rates in the euphotic (0.5 m) and aphotic layers (60 m) of Lake Nam Co. Viruses were abundant in both layers (Fig. S5), with VLP abundance significantly higher in the aphotic layer (3.4 ± 0.4 × 10^6^ VLPs ml^−1^) compared to the euphotic layer (1.9 ± 1.0 × 10^6^ VLPs ml^−1^, *P* < .05) (Fig. 2A). In contrast, bacterial abundance was greater in the euphotic layer (1.3 ± 0.2 × 10^6^ cells ml^−1^) than in the aphotic layer (0.9 ± 0.02 × 10^6^ cells ml^−1^, *P* < .05) (Fig. 2A). A similar trend appeared in heterotrophic bacterial production, which was elevated in the euphotic layer (6.4 × 10^3^ cells ml^−1^ h^−1^) compared to the aphotic layer (4.8 × 10^3^ cells ml^−1^ h^−1^, Fig. S6A). Virus-to-bacterium ratio was significantly higher in the aphotic layer (3.9 ± 0.6) than in the euphotic layer (1.5 ± 0.3, *P* < .05) (Fig. S6B).

Lytic and lysogenic viral production did not significantly differ between two layers (dark incubations, Fig. 2B), and the frequency of lytic and lysogenic infection cells showed similar vertical patterns (dark incubations, Fig. 2C). Viral decay rates, however, were significantly higher in the euphotic layer (5.5 ± 1.5%) compared to the aphotic layer (2.1 ± 0.04%, *P* < .05) (dark incubations, Fig. 2D).

### Vertical pattern of bacterial and viral diversity

**Figure 3:**
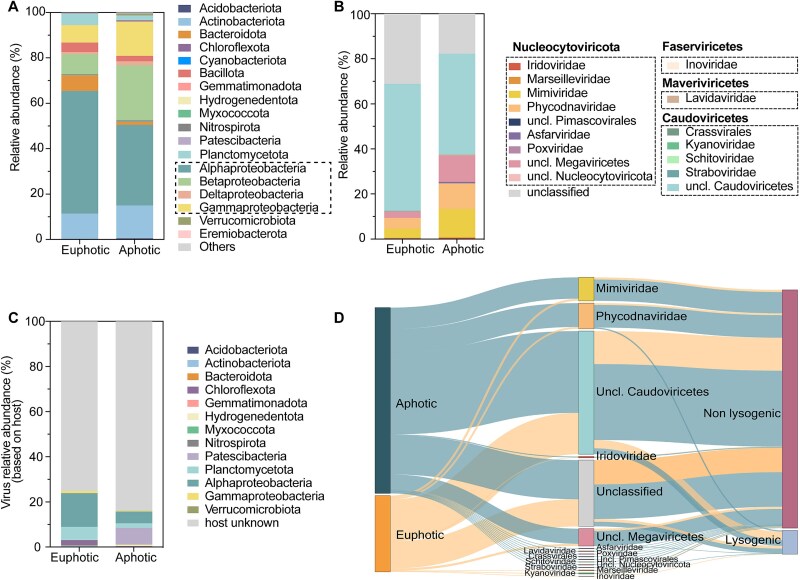
Viral and bacterial community composition and host-virus associations in euphotic and aphotic water layers. (A) Bacterial community composition in euphotic and aphotic layers based on read-level classification using Kraken2. (B) Taxonomic composition of classified vOTUs in euphotic and aphotic layers. (C) Relative abundance of viruses associated with predicted host types in each layer. (D) Sankey diagram of virus-host associations and life strategies in each water layer.

Metagenomic sequencing revealed distinct bacterial and viral communities between the euphotic and aphotic layers. The euphotic layer was dominated by Alphaproteobacteria (54.0%), followed by Actinobacteriota (11.1%), Betaproteobacteria (8.9%), and Gammaproteobacteria (7.7%), whereas the aphotic layer showed reduced Alphaproteobacteria (35.5%) and increased proportions of Betaproteobacteria (24.3%), Gammaproteobacteria (14.9%), and Actinobacteriota (14.4%) (Fig. 3A). Additionally, Taxa such as Bacteroidota, Bacillota, and Planctomycetota were comparatively depleted in the aphotic layer (Fig. 3A).

Viral diversity also showed clear vertical stratification. A total of 872 viral contigs ≥10 kb clustering into 849 vOTUs, with 611 vOTUs in the aphotic layer and 260 in the euphotic layer, and only 22 shared vOTUs (Fig. S7). In the euphotic layer, 68.9% of the vOTUs were classified, with unclassified *Caudoviricetes* comprising 56.3%. The aphotic layer had 82.3% classified vOTUs, predominantly unclassified Caudoviricetes (44.6%) and Megaviricetes (36.4%), with major families including *Mimiviridae* (12.6%) and *Phycodnaviridae* (11.4%) (Fig. 3B). Predicted virus-host associations also varied: 24.8% of vOTUs in the euphotic layer were associated with hosts, predominantly Alphaproteobacteria, Planctomycetes, and Chloroflexota, while in the aphotic layer, only 16.1% of vOTUs were linked with hosts, primarily Patescibacteria, Alphaproteobacteria and Planctomycetota (Fig. 3C, D). Additionally, the proportion of lysogenic vOTUs was higher in the euphotic layer (15.9%) compared to the aphotic layer (6.5%) (Figs. S8A, B).

**Figure 4:**
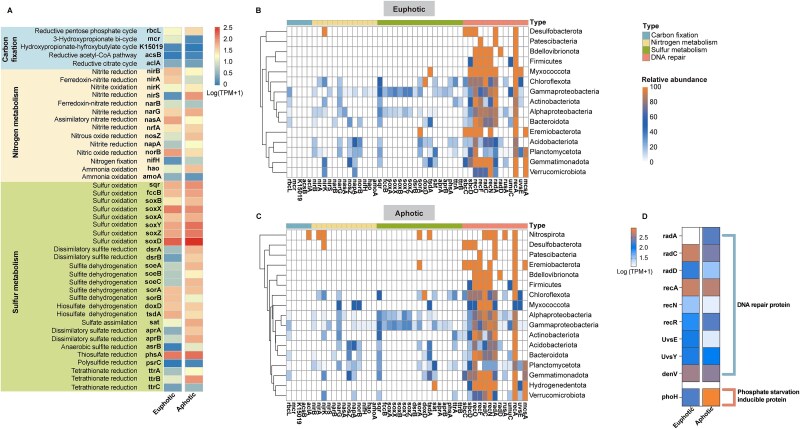
Distribution and abundance of functional genes across euphotic and aphotic layers. (A) Abundance of bacterial functional genes involved in carbon fixation, nitrogen metabolism, and sulfur metabolism within euphotic and aphotic layers. (B, C) Distribution of selected key functional genes in the obtained MAGs from (B) euphotic and (C) aphotic water layers, covering carbon fixation, nitrogen and sulfur metabolism, and DNA repair pathways. (D) Abundance of viral genes associated with DNA repair proteins and phosphate starvation-inducible proteins detected within vOTUs.

Functional gene profiling further highlighted depth-related divergence. Genes involved in sulfur metabolism were prevalent in the aphotic layer (Fig. 4A). Carbon fixation genes related to the reductive pentose phosphate cycle and the 3-Hydroxypropionate bi-cycle were dominated in the euphotic layer, while reductive pentose phosphate cycle gene dominated the aphotic layer (Fig. 4A). Among the 160 reconstructed MAGs, key metabolic genes involved in carbon, nitrogen and sulfur metabolism, as well as DNA repair, showed unique distribution across water layer. Notably, the *aclA* gene was only detected in Nitrospirota, exclusive to the euphotic layer (Fig. 4B, C).

Viral AMGs related to cellular processes, environmental information processing, human diseases, and general metabolism were more abundant in the euphotic layer, whereas those associated with environmental adaptation (organismal systems) and genetic information processing were more prevalent in the aphotic layer (Fig. S9). Furthermore, DNA repair genes in vOTUs were more abundant in the euphotic layer, whereas the phosphate starvation-inducible protein gene (*phoH*) was more prevalent in the aphotic layer (Fig. 4D).

### Effects of solar radiation on viral production and decay rates

To evaluate the effects of solar radiation on viral production and decay, we conducted parallel experiments comparing viral dynamic parameters under dark and light-transmitting incubations during both diel and vertical investigations.

**Figure 5:**
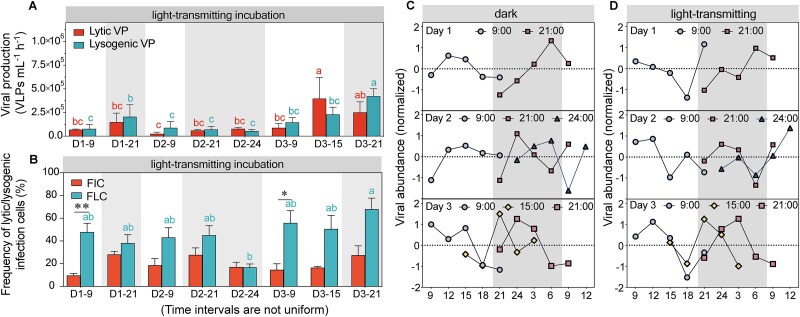
(A) Lytic and lysogenic viral production (VP) under light-transmitting incubations. (B) Frequency of lytic and lysogenic infected cells (FIC and FLC) under light-transmitting. (C, D) Viral abundance over time in the virus dilution approach in (C) dark incubation and (D) light-transmitting incubation. Shaded areas represent night.

**Figure 6:**
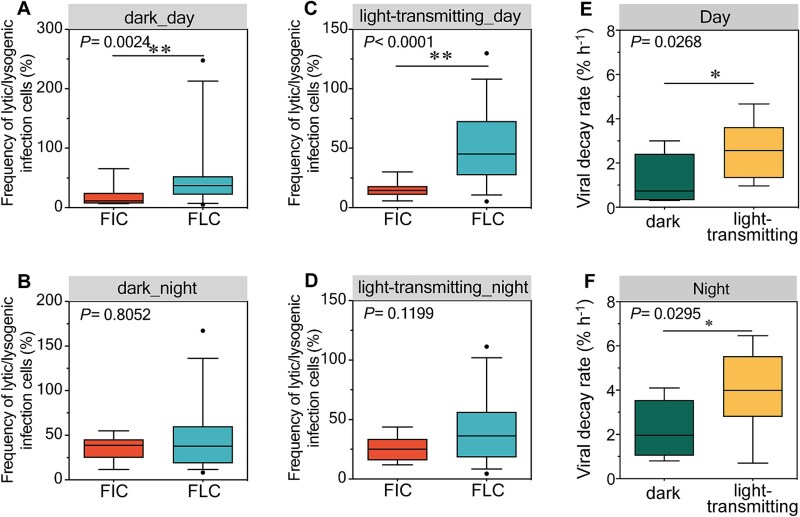
(A–D) Detailed comparisons of FIC and FLC across conditions: Daytime (A) and nighttime (B) comparisons of FIC and FLC in dark incubations; daytime (C) and nighttime (D) comparisons of FIC and FLC in light-transmitting incubations. (E–F) Comparative analysis of VD between dark and light-transmitting incubations across (E) day, and (F) night.

During diel variation experiments at the lake shore, llytic and lysogenic productions, as well as infection frequencies, under light-transmitting conditions followed similar fluctuation patterns to those observed under dark conditions, with no pronounced diel variation (Fig. 5A, B). Viral abundance remained stable at night across treatments but diverged during daytime, with differences emerging between light and dark incubations (Fig. 5C, D). Overall, viral production rates did not significantly differ between treatments during either period (Fig. S10). However, during daylight hours, the frequency of lysogenic infections significantly exceeded that of lytic infections, particularly under light-transmitting conditions, while no such difference was observed at night (Fig. 6A–D). Viral decay rates were higher under light-transmitting conditions (0.8%–6.0%) compared to dark incubations (0.4%–3.8%), regardless of day or night periods (*P* < .05, Figs. 1D and 6E, F).

On the vertical scale, within the euphotic layer, light-transmitting conditions significantly elevated lytic and lysogenic production, as well as lysogenic infection frequency, compared to dark incubations, reaching 1.0 ± 0.6 × 10^5^ VLPs ml^−1^ h^−1^, 1.5 ± 0.4 × 10^5^ VLPs ml^−1^ h^−1^, and 177.6 ± 112.9%, respectively (*P* < .05). Conversely, these parameters were similar between the two incubations in the aphotic layer, with values of 2.2 × 10^4^ VLPs ml^−1^ h^−1^, 6.9 × 10^4^ VLPs ml^−1^ h^−1^, and 29.5% under light-transmitting incubations, respectively (Fig. 2B, C). Interestingly, within the euphotic layer, VD were significantly higher in dark incubations compared to light-transmitting conditions (*P* < .05, Fig. 2D).

## Discussion

### Synergistic regulation of diel variation of surface viral ecology by solar radiation and stream input

Previous studies found solar radiation as a critical factor in viral decay and infectivity loss in surface waters [54, 55]. Given the Tibetan Plateau’s intense solar exposure [17], we hypothesized that it would be the dominant regulator of viral ecology in Tibetan lakes. Surprisingly, no clear diel variations in VLP abundance, viral production, or VD were observed. Although autotrophic bacterial abundance showed a diel pattern, heterotrophic bacteria displayed more stability and close correlation with salinity, turbidity, pH, and Chl*-a*, indicating they are more influenced by physicochemical factors than by PAR (Fig. S4). Previous studies linked the diel fluctuations of turbidity, pH, TDS, salinity, and ion concentrations in Lake Nam Co to glacial meltwater and precipitation-driven stream inflows [56]. Daytime stream inflows elevate turbidity, low pH, and introduce unique ionic compositions [56, 57], which appear to mitigate solar radiation’s impacts on viral ecology, fostering a stable VLP abundance across the diel cycle.

In high-radiation environment, bacteria in Tibetan lakes may adapt by producing carotenoids [58] or forming biofilms [59]. Elevated turbidity from meltwater during the day [60] further scatters solar radiation, thereby reducing its impact on bacterial and viral populations [61]. This buffering effect likely contributes to the observed diel stability of heterotrophic bacterial populations, which in turn stabilizes viral production that is closely tied to host abundance [62]. Additionally, the low abundance of autotrophic bacteria in oligotrophic alpine lakes [63] limits the nutrient availability for heterotrophic bacteria growth, further minimizing influence from autotrophic bacterial diel fluctuations on heterotrophic bacterial growth and reproduction [64, 65].

Dark and light-transmitting incubation experiments revealed that solar radiation modifies viral infection strategies by promoting lysogenic phage production and enhancing viral survival under sunlight. Under dark incubations, VLP abundance increased rapidly, indicating lytic activity, whereas under light-transmitting conditions, VLP abundance peaks appeared later, possibly to avoid sunlight-induced DNA damage (Figs. 5C, D) [28]. This temporal shift of viral infection strategy did not alter the overall VLP standing stock (Figs. 1B and 5A), likely due to an increased burst size (BS) compensating for lower lytic infection frequency under high radiation (Fig. S11). This is supported by a positive relationship between virus-to-bacterium ratio and PAR (Fig. S4), suggesting that solar radiation boosts viral release per lysis event, thereby sustaining high levels of lytic production despite a lower frequency of lytic infection cells.

Parallel incubation experiments also revealed that solar radiation contributes to viral decay by damaging viral DNA, proteins, and structural components, thereby reducing their infectivity [9]. However, diel stability in viral decay rate suggests that stream-introduced turbidity might play a compensatory role, attenuating up to 25% of the radiation and mitigating its damaging effects on viruses (Table S2) [61]. This buffering effect likely explains the observed diel stability in VD and VLP abundance, reinforcing the importance of stream inputs in moderating solar effects on viral decay.

### Solar radiation as a key factor influencing vertical variation in viral ecology

Distinct vertical variations in bacterial and viral abundance, composition, and viral dynamics were observed within Lake Nam Co (Figs. 2, 3), likely driven by solar radiation gradients along the depth profile. PAR decreases sharply with depth, reaching only 0.2% at 30 m [66]. Bacterial abundance and production rates in the euphotic layer are significantly higher than in the aphotic layer (60 m), further suggesting adaptions to intense solar radiation that allow for greater surface-level bacterial productivity (Fig. S6A). In the euphotic zone, stronger solar radiation likely promotes lysogenic viral infections, as suggested by elevated frequency of lysogenic viral infection (Fig. 2C) and increased abundance of lysogenic viruses (Fig. 3D). Despite lysogeny’s predominance, higher host abundance and activity in this zone sustains viral production at levels comparable to the aphotic layer. However, VD in the euphotic layer are significantly elevated due to damage from intense solar radiation (Fig. 2D) [9], resulting in lower VLP abundance compared to the aphotic layer [67].

Solar inhibition of chlorophyll-*a* synthesis and suppression of algae reproduction in surface water also likely contributes to vertical variation, with the maximum Chl*-a* concentration occurring at around 60 meters, where light inhibition is lessened due to thermal stratification [68, 69]. This increased Chl*-a* concentration in the aphotic layer supported substantial plankton and algae populations [69], explaining the observed abundance of algal viruses like Nucleocytoviricota (such as *Miniviridae*, *Phycodnaviridae*) in the aphotic layer (Fig. 3B). Functional diversity differences in bacterial and viral communities between the euphotic and aphotic layers further support this depth-dependent adaptation (Fig. 4). Notably, bacterial genes associated with DNA repair are comparably abundant in both layers (Figs. 4B, C, Fig. S12), likely reflecting bacterial adaptations like biofilm formation and carotenoid synthesis that mitigate radiation-induced DNA damage [59]. In contrast, viral genes linked to DNA repair are more prevalent in the euphotic layer (Fig. 4D), suggesting viral strategies to cope with solar-induced DNA damage [55, 70].

## Conclusion

**Figure 7:**
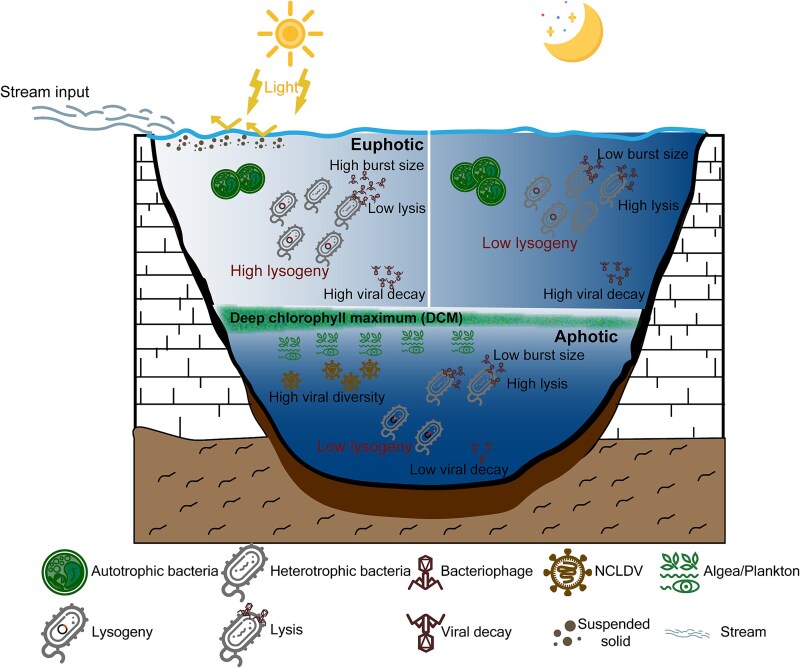
Schematic overview of diel and vertical virus-host interactions in Lake Nam Co. In surface waters, FLC is higher during the day, while FIC is lower. At night, FIC increases, yet overall viral production remains stable due to a reduced viral burst size. Streamborne suspended solids partially absorb and scatter solar radiation, moderating the viral decay rate and minimizing day-night differences. In the aphotic layer, high FIC and low FLC prevail, with VD significantly reduced in the absence of solar radiation, enabling viral population accumulation. The deep chlorophyll maximum layer, enriched with nucleocytoplasmic large DNA viruses, supports high viral diversity.

Our study provides new insights into the diel and vertical dynamics of viral ecology in the alpine lake Nam Co, highlighting the unique sensitivity of viruses to the intense solar radiation on the Tibetan Plateau. Our findings indicate that elevated solar radiation promotes lysogenic phage production, prolongs the latent period, and accelerates viral degradation (Fig. 7). These responses likely influence virus-mediated carbon release through lysis and decay, with implications for biogeochemical cycling in alpine lake ecosystems. Additionally, stream inflows play a critical role in tempering solar radiation’s effects on viral ecology, resulting in minimal diel variations in viral dynamics, particularly near the lake shore (Fig. 7). This study reveals the coordinated regulation of viral processes by solar radiation and glacial melt-driven stream inflows, providing valuable insights into viral contributions to the biogeochemical cycles in a high-altitude lake ecosystem.

## Supplementary Material

Supplymentary_Information_ycaf129

## Data Availability

Raw sequence data are deposited in the NCBI archive under accession number PRJNA1197720. Other data that support the findings of this study are available from the corresponding author upon reasonable request.
