# Supplementary material for: Solar radiation shapes viral ecology in an alpine lake at 4700 meters
Source: ISME Commun. 2025 Aug 8;5(1):ycaf129. doi: 10.1093/ismeco/ycaf129 (PMC12602380; doi:10.1093/ismeco/ycaf129)
Supplement: Supplymentary_Information_ycaf129 [file supplymentary_information_ycaf129.pdf]

Supplementary Figures

Solar Radiation Shaping Viral Ecology in an Alpine Lake at 4,700 Meters

Lin Zang[1,7], Yongqin Liu[1,2,7]*, Nianzhi Jiao[3], Lanlan Cai[4], Wei Wei[5], Xiaowei Chen[3], Yuying Chen[2], Keshao Liu[1], Rui Zhang[6]*

[1] State Key Laboratory of Tibetan Plateau Earth System, Environment and Resources (TPESER), Institute of Tibetan Plateau Research, Chinese Academy of Sciences, Beijing 100101, China;

[2] Center for the Pan-Third Pole Environment, Lanzhou University, Lanzhou 730000, China;

[3] State Key Laboratory of Marine Environmental Science, College of Ocean and Earth Sciences, Fujian Key Laboratory of Marine Carbon Sequestration, Xiamen University, Xiamen 361102, China;

[4] Earth, Ocean and Atmospheric Sciences Thrust, The Hong Kong University of Science and Technology (Guangzhou), Guangzhou 511453, China;

[5]Research Center for Environmental Ecology and Engineering, School of Environmental Ecology and Biological Engineering, Wuhan Institute of Technology, Wuhan 430205, China;

[6] Archaeal Biology Center, Synthetic Biology Research Center, Shenzhen Key Laboratory of Marine Microbiome Engineering, Key Laboratory of Marine Microbiome Engineering of Guangdong Higher Education Institutes, Institute for Advanced Study, Shenzhen University, Shenzhen 518055, China;

[7] University of Chinese Academy of Science, Beijing 100101, China;

*Corresponding author. Email: Yongqin Liu, yql@lzu.edu.cn; Rui Zhang, ruizhang@szu.edu.cn

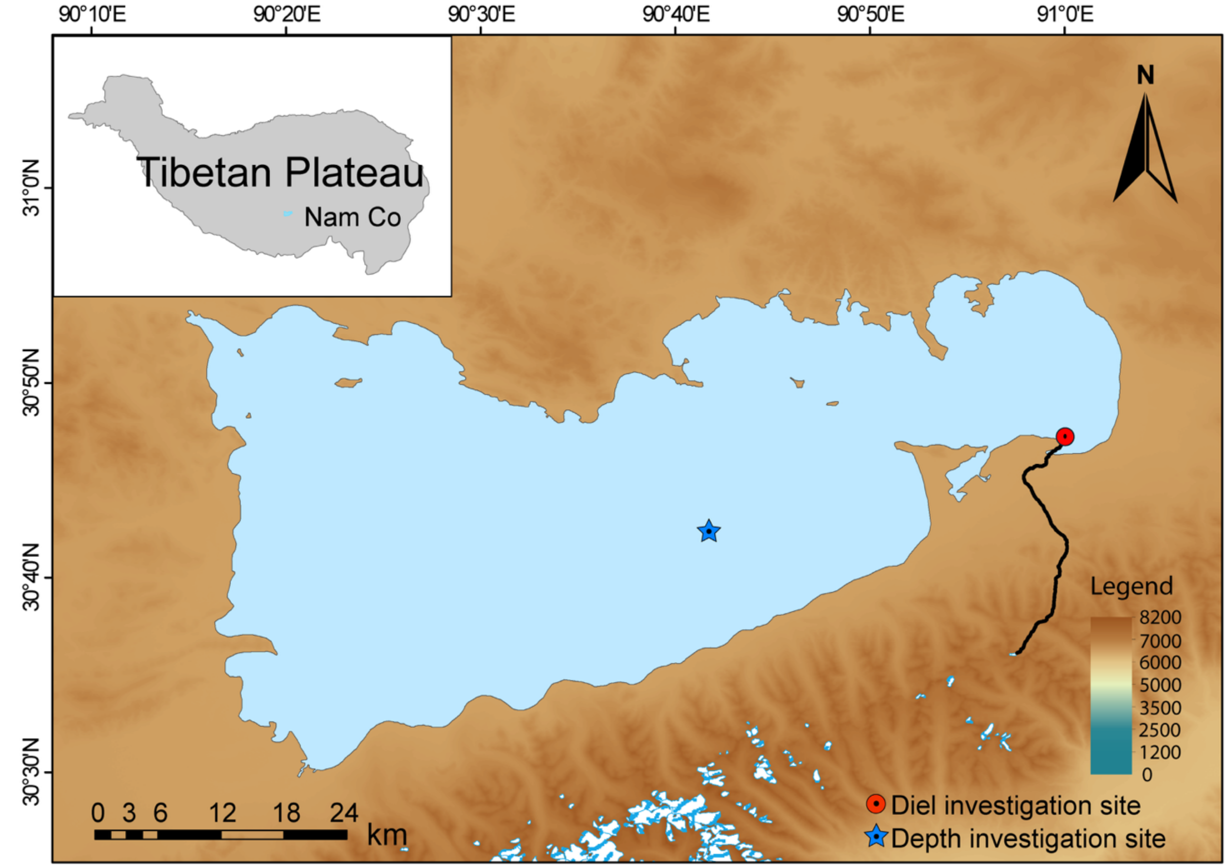

Fig. S1 Geographic overview of Nam Co on the Tibetan Plateau and sampling sites.

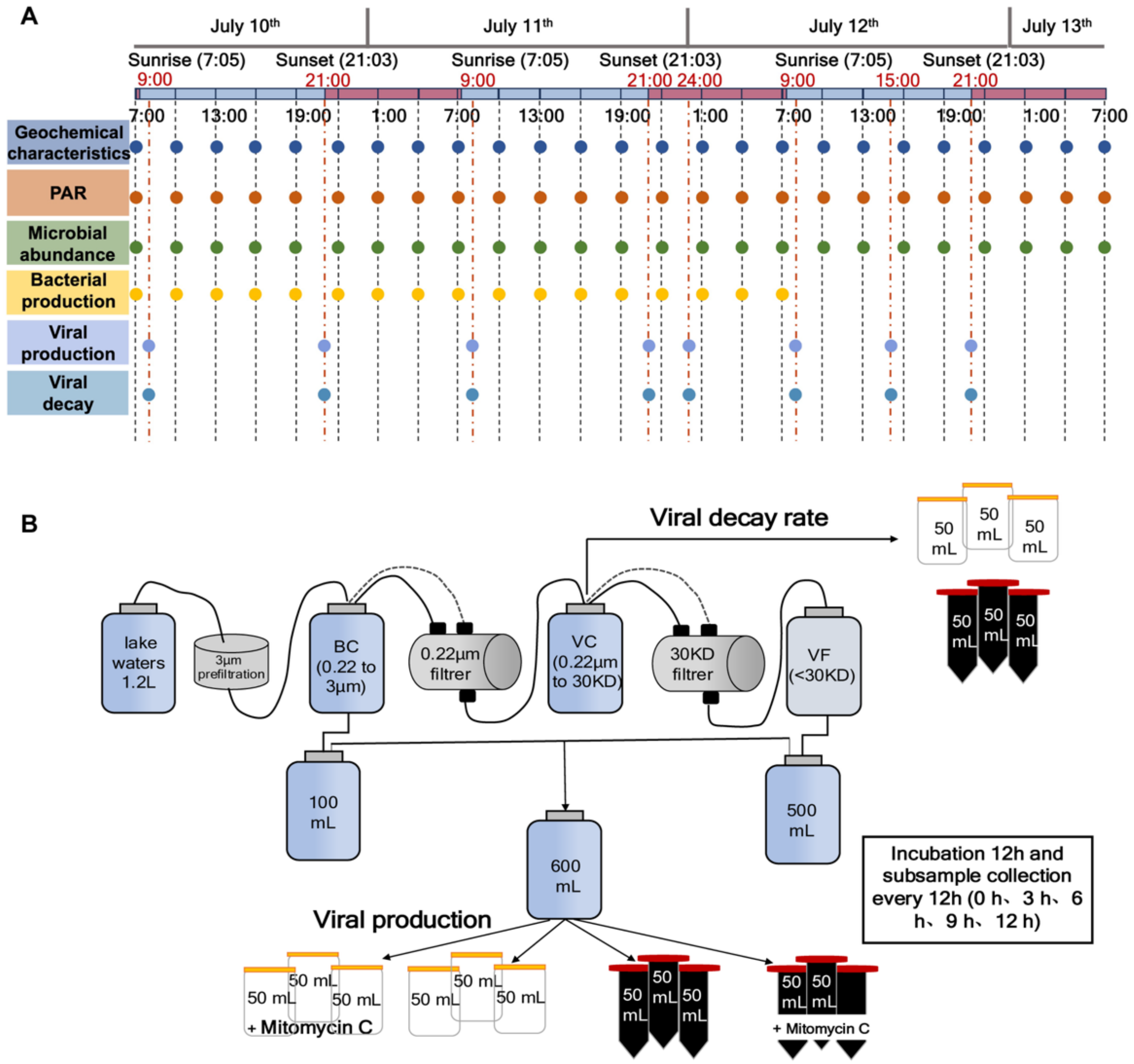

Fig. S2 Overview of field investigation. A Schematic diagram of time series sampling (dots represent sampling time points); **B** Overview Diagram of field investigation on viral dynamic.

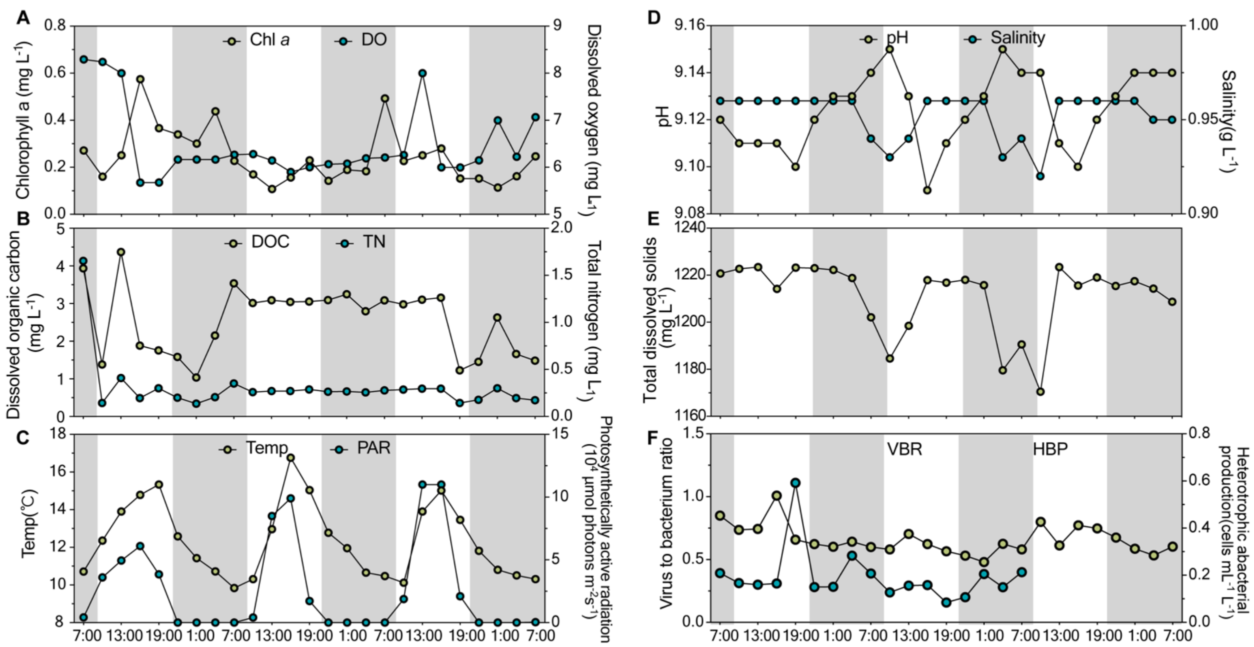

Fig. S3 Variability in geochemical characteristics A Variability in Chlorophyll *a* and dissolved oxygen concentrations; **B** Variability in dissolved organic carbon and total nitrogen concentrations; **C** Variability in temperature and photosynthetically active radiation; D Variability in pH and salinity; **E** Variability in total dissolved solids; **F** Variability in virus to bacterium ratio (VBR) and heterotrophic bacterial production (HBP).

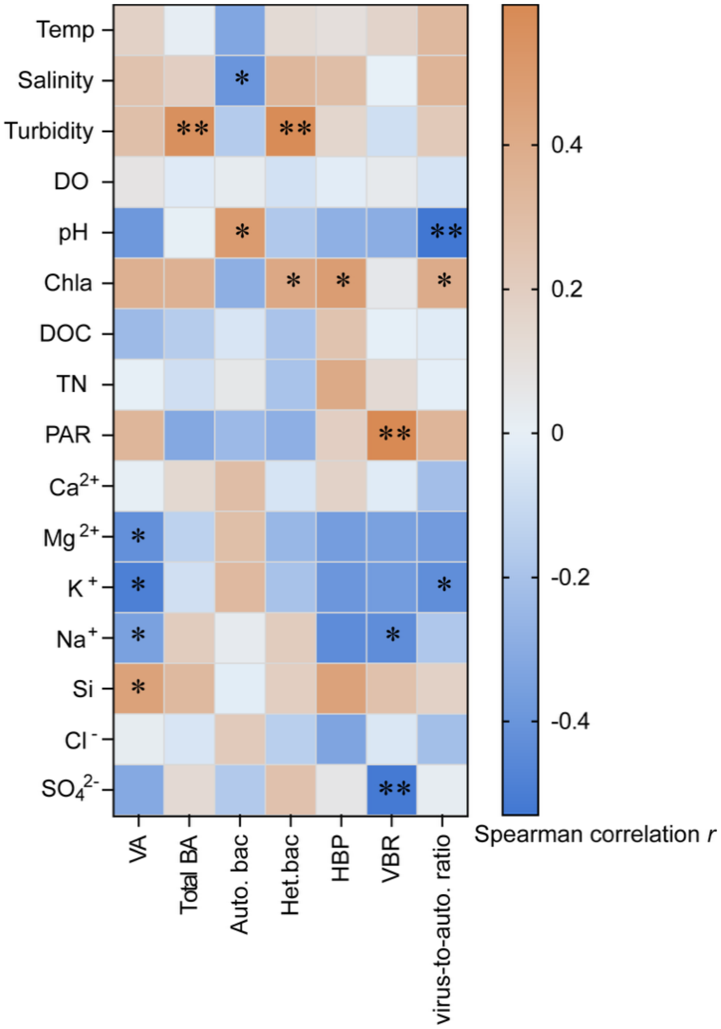

Fig. S4 Spearman correlation (rs) analysis of VLP abundance (VA), total bacterial abundance (Total bac), autotrophic bacterial abundance (Auto. bac), heterotrophic bacterial abundance (Het. bac), heterotrophic bacterial production (BP), virus-to-heterotrophic bacterium ratio (VBR), virus-to-autotrophic bacteria ratio and geochemical characteristics.

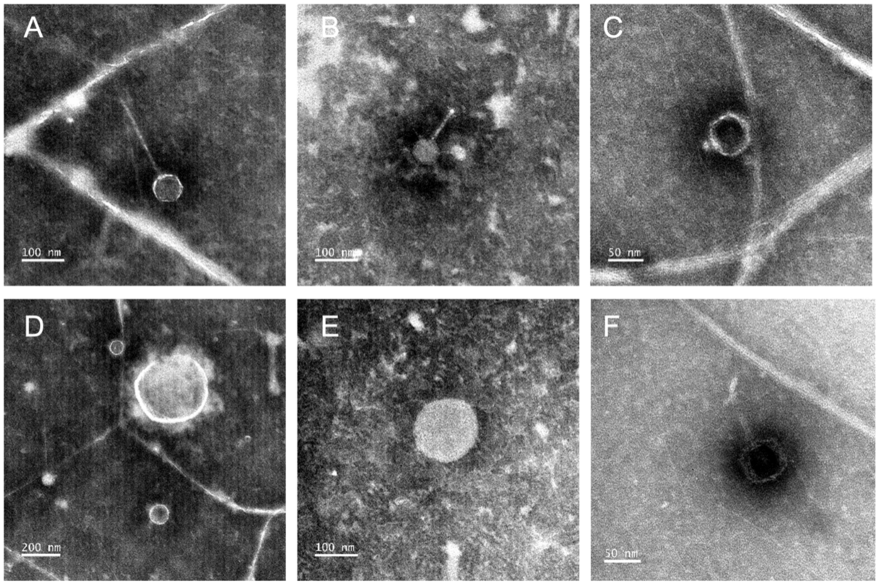

Fig. S5 Transmission electron micrographs of virus-like particles in the euphotic and aphotic water layers.

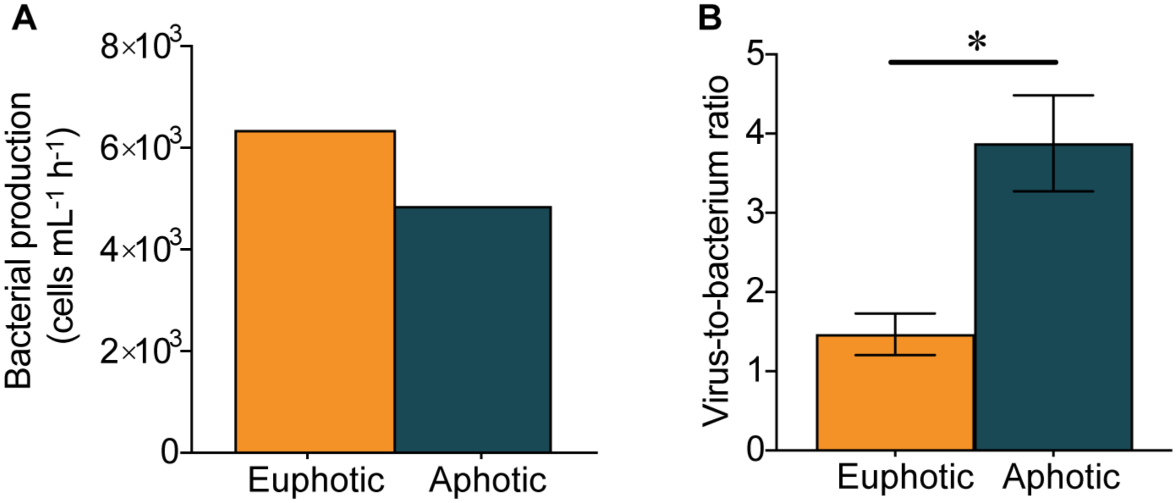

Fig. S6 Bacterial production (A) and virus-to-bacterium ratio (B) in the euphotic and aphotic water layers.

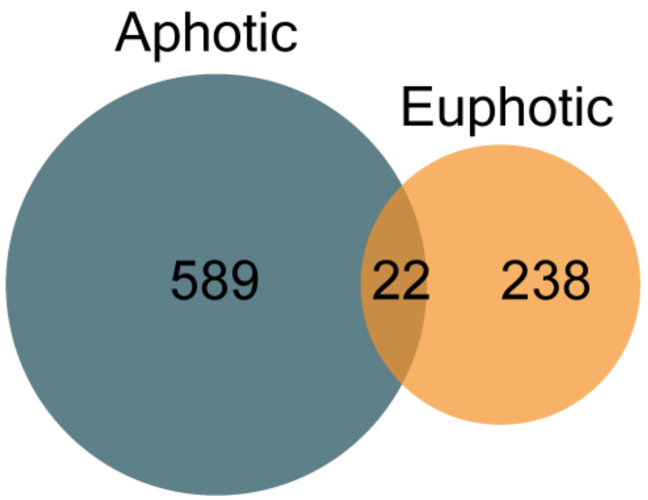

Fig. S7 Venn diagram of viral operational taxonomic units (vOTUs) shared between euphotic and aphotic layers.

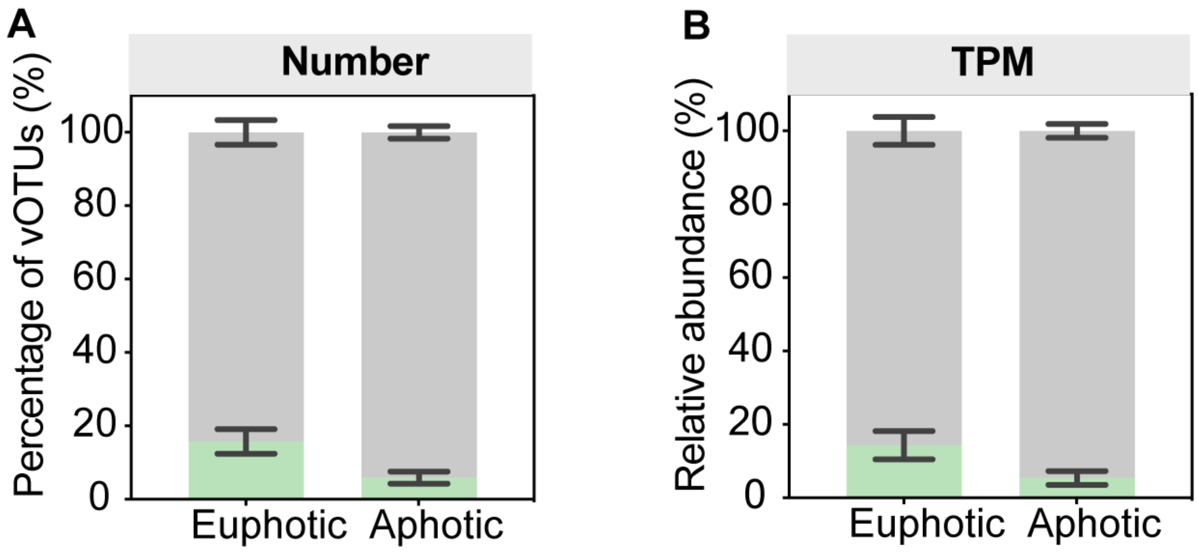

Fig. S8 Proportion (A) and relative abundance (B) of lysogenic vOTUs in the euphotic and aphotic water layers.

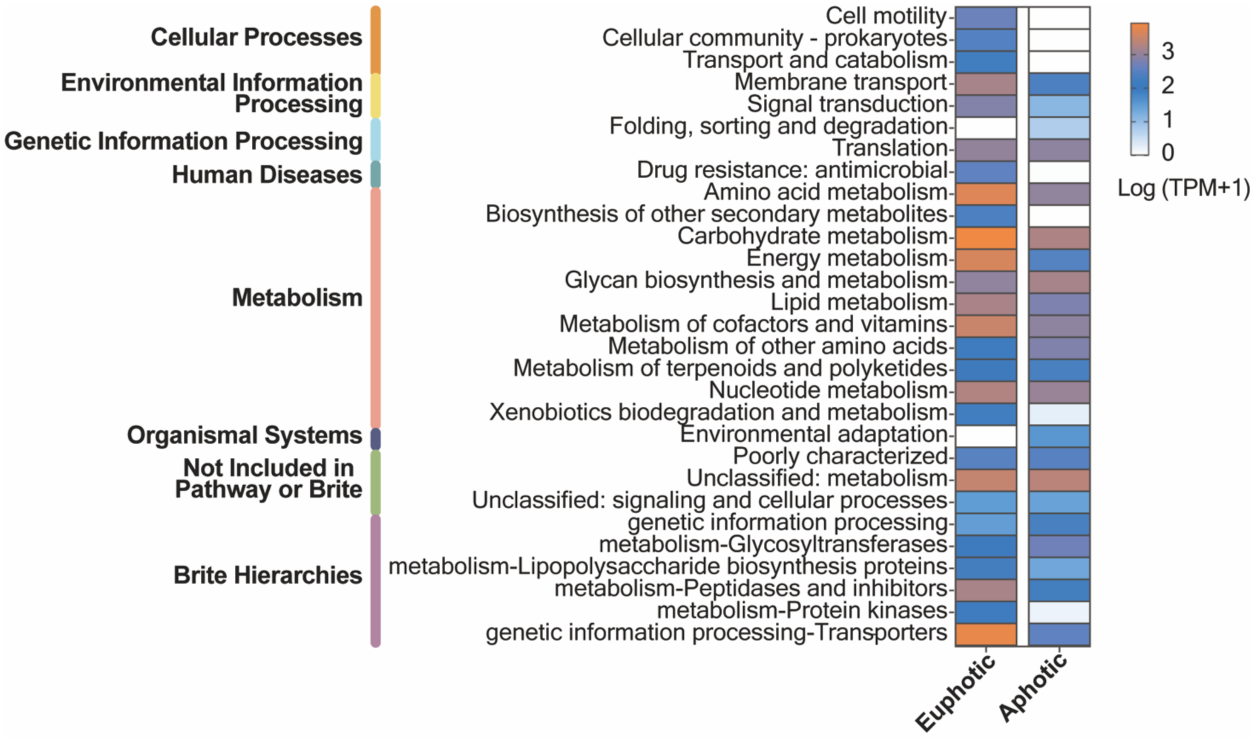

Fig. S9 Abundance of auxiliary metabolic genes (AMGs) detected in vOTUs.

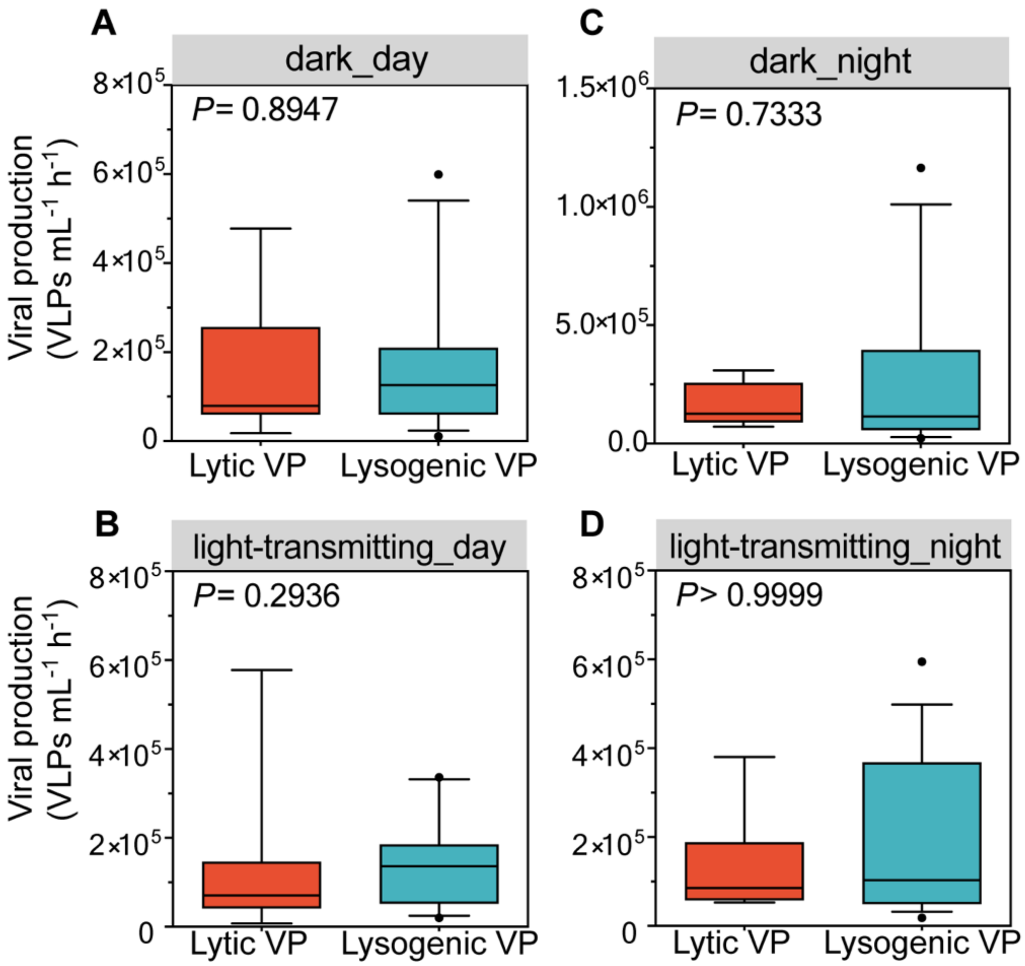

Fig. S10 Detailed comparisons of lytic and lysogenic viral production (VP) across conditions: daytime lytic VP and lysogenic VP in **(A)** dark and **(B)** light-transmitting incubations; nighttime lytic VP and lysogenic VP in **(C)** dark and **(D)** light-transmitting incubations.

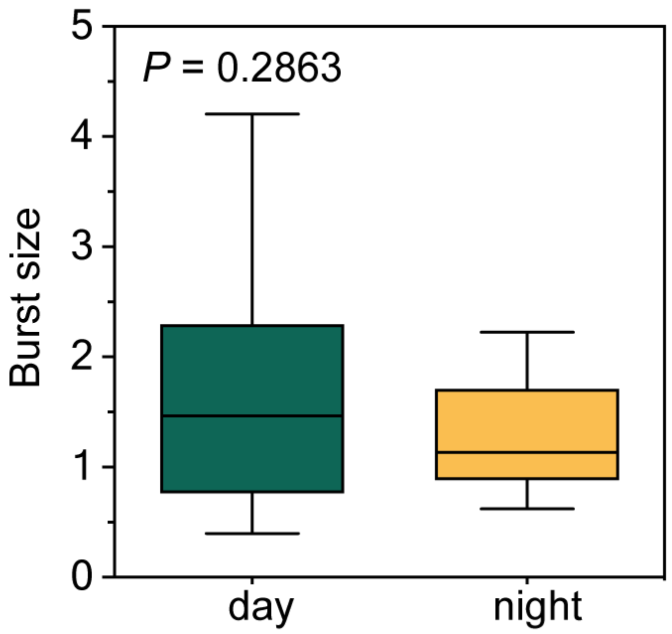

Fig. S11 Comparison of viral burst size during day and night.

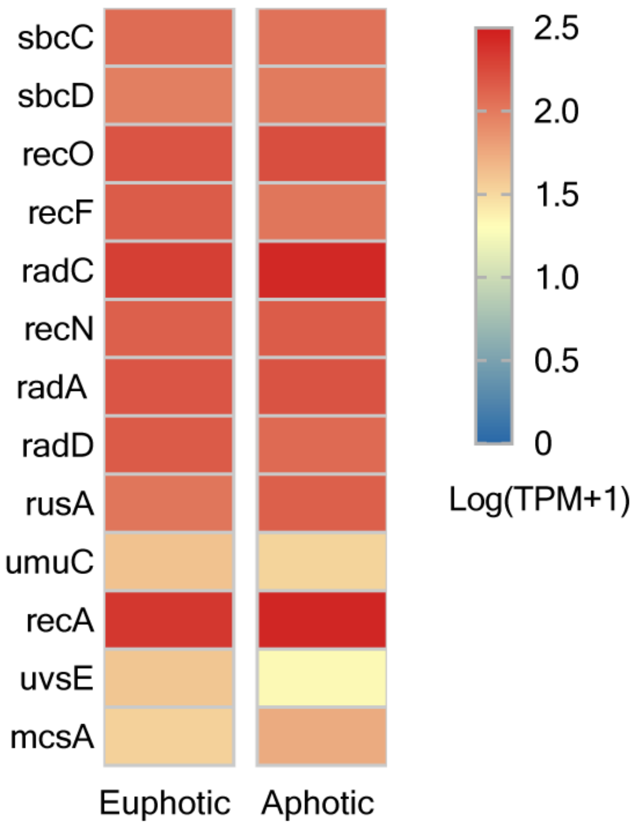

Fig. S12 Abundance of bacterial genes associated with DNA repair.

Table S1 Variability in geochemical characteristics

Time	Temp (°C)	Salinity (g L^{-1})	Turbidity (FNU)	pH	PAR (10^4 μmol photons m^{-2} s^{-1})	DO	Chl-*a*	DOC	TN	Ca^{2+}	Mg^{2+}	K^+	Na^+	Cl^-	NO_3^-	SO_4^{2-}
						(mg L^{-1})										
7:00	10.71	0.96	20.10	9.12	0.42	8.29	0.27	3.94	1.65	7.86	73.75	32.60	258.76	70.17	n.a.	179.27
10:00	12.36	0.96	3.45	9.11	3.60	8.24	0.16	1.38	0.14	7.65	74.82	33.73	264.17	70.64	n.a.	187.38
13:00	13.90	0.96	29.37	9.11	4.95	8.00	0.25	4.37	0.41	7.93	74.15	33.16	260.32	70.21	n.a.	185.55
16:00	14.79	0.96	80.46	9.11	6.10	5.67	0.57	1.88	0.20	8.06	73.74	33.67	264.66	69.31	n.a.	185.18
19:00	15.34	0.96	45.67	9.10	3.85	5.68	0.37	1.76	0.30	7.84	47.63	22.96	186.12	43.50	n.a.	119.42
22:00	12.58	0.96	36.55	9.12	0.00	6.17	0.34	1.58	0.20	8.22	75.64	34.27	267.37	72.17	n.a.	188.67
1:00	11.43	0.96	29.74	9.13	0.00	6.16	0.30	1.04	0.14	8.30	75.19	34.39	266.64	72.19	n.a.	191.48
4:00	10.72	0.96	30.28	9.13	0.00	6.17	0.44	2.15	0.21	8.30	76.60	34.29	264.47	72.63	n.a.	192.69
7:00	9.84	0.94	21.07	9.14	0.01	6.27	0.23	3.54	0.35	8.17	74.61	33.74	260.50	71.00	0.03	187.29
10:00	10.31	0.93	20.77	9.15	0.41	6.28	0.17	3.01	0.26	7.83	75.09	33.65	259.64	69.48	n.a.	184.41
13:00	12.97	0.94	9.09	9.13	8.50	6.14	0.11	3.09	0.27	8.04	75.47	33.93	260.99	64.68	n.a.	167.58
16:00	16.76	0.96	5.43	9.09	9.90	5.90	0.16	3.05	0.27	8.00	76.52	34.50	261.85	72.11	n.a.	190.91
19:00	15.04	0.96	10.09	9.11	1.72	6.00	0.23	3.06	0.29	7.64	77.00	34.50	265.27	70.93	n.a.	190.66
22:00	12.77	0.96	10.36	9.12	0.00	6.06	0.14	3.09	0.26	7.75	76.71	34.46	266.97	71.37	n.a.	189.90
1:00	11.95	0.96	18.93	9.13	0.00	6.08	0.19	3.25	0.27	8.17	75.30	34.52	264.56	71.43	n.a.	189.95
4:00	10.66	0.93	12.18	9.15	0.00	6.19	0.18	2.80	0.26	8.09	73.57	31.11	240.47	64.30	n.a.	168.84
7:00	10.47	0.94	18.22	9.14	0.01	6.21	0.49	3.09	0.28	8.65	76.12	33.81	260.61	70.11	n.a.	189.81
10:00	10.12	0.92	26.43	9.14	1.88	6.26	0.23	2.98	0.29	8.31	71.58	32.44	251.83	211.23	12.89	185.38
13:00	13.90	0.96	29.37	9.11	11.00	8.00	0.25	3.11	0.30	8.62	75.55	33.32	258.55	78.70	4.06	167.91
16:00	15.02	0.96	17.37	9.10	11.00	6.00	0.28	3.16	0.30	8.07	77.09	34.57	263.91	80.94	2.91	178.34
19:00	13.46	0.96	9.16	9.12	2.10	5.99	0.15	1.23	0.14	8.36	77.05	34.67	263.98	87.17	1.03	179.33
22:00	11.81	0.96	12.56	9.13	0.00	6.15	0.15	1.45	0.18	8.14	69.20	32.61	253.29	136.55	12.76	179.60
1:00	10.80	0.96	12.97	9.14	0.00	7.00	0.11	2.63	0.30	8.39	75.90	34.79	267.94	87.06	11.21	183.17
4:00	10.51	0.95	44.21	9.14	0.00	6.22	0.16	1.66	0.20	8.03	75.68	34.43	264.78	104.48	22.76	181.06
7:00	10.31	0.95	20.69	9.14	0.04	7.07	0.25	1.48	0.17	8.49	75.69	34.34	264.38	101.07	17.39	182.26
Euphotic	12.94	1.05	0.47	9.30	n.a.	11.08	0.15	n.a.	n.a.	n.a.	n.a.	n.a.	n.a.	n.a.	n.a.	n.a.
Aphotic	3.58	1.05	14.58	9.35	n.a.	12.56	1.80	n.a.	n.a.	n.a.	n.a.	n.a.	n.a.	n.a.	n.a.	n.a.

Temp: temperature; PAR: photosynthetically active radiation; DO: dissolved oxygen; Chl a: Chlorophyll a; DOC: dissolved organic carbon; TN: total nitrogen

Table S2 Spearman correlation (rs) analysis of *in situ* viral production and viral decay rate with geochemical characteristics.

	Lytic viral production	Lysogenic viral production	Viral decay rate
Temp	0.67	0.26	0.26
Salinity	0.38	0.25	0.38
Turbidity	-0.52	-0.05	-0.55*
pH	-0.60	-0.26	-0.16
PAR	-0.29	0.12	-0.36
DO	-0.79*	-0.47	-0.48
Chl a	0.07	0.30	-0.35
DOC	-0.61	-0.40	-0.55
TN	-0.47	-0.41	-0.87**
Ca^{2+}	0.05	0.52	0.12
Mg^{2+}	0.74*	0.40	0.74*
K^{+}	0.71	0.43	0.78*
Na^{+}	0.36	-0.02	0.71
Cl^{-}	0.43	0.26	0.62
SO_4^{2-}	-0.17	-0.50	0.17
